# Functional Connectivity-Based Modelling Simulates Subject-Specific Network Spreading Effects of Focal Brain Stimulation

**DOI:** 10.1007/s12264-018-0256-0

**Published:** 2018-07-24

**Authors:** Xiaoyu Chen, Chencheng Zhang, Yuxin Li, Pei Huang, Qian Lv, Wenwen Yu, Shengdi Chen, Bomin Sun, Zheng Wang

**Affiliations:** 1 Institute of Neuroscience, State Key Laboratory of Neuroscience, Chinese Academy of Sciences Center for Excellence in Brain Science and Intelligence Technology, Shanghai Institute for Biological Sciences, Chinese Academy of Sciences, Shanghai, 200031 China; 2 University of Chinese Academy of Sciences, Beijing, 100049 China; 3 Department of Functional Neurosurgery, Ruijin Hospital, Shanghai Jiao Tong University School of Medicine, Shanghai, 200025 China; 4 Department of Radiology, Huashan Hospital, Fudan University, Shanghai, 200040 China; 5 Department of Neurology, Ruijin Hospital, Shanghai Jiao Tong University School of Medicine, Shanghai, 200025 China

**Keywords:** Brain stimulation, Functional connectivity, Whole-brain modeling, Parkinson’s disease, Individual variability

## Abstract

Neurostimulation remarkably alleviates the symptoms in a variety of brain disorders by modulating the brain-wide network. However, how brain-wide effects on the direct and indirect pathways evoked by focal neurostimulation elicit therapeutic effects in an individual patient is unknown. Understanding this remains crucial for advancing neural circuit-based guidance to optimize candidate patient screening, pre-surgical target selection, and post-surgical parameter tuning. To address this issue, we propose a functional brain connectome-based modeling approach that simulates the spreading effects of stimulating different brain regions and quantifies the rectification of abnormal network topology in silico. We validated these analyses by pinpointing nuclei in the basal ganglia circuits as top-ranked targets for 43 local patients with Parkinson’s disease and 90 patients from a public database. Individual connectome-based analysis demonstrated that the globus pallidus was the best choice for 21.1% and the subthalamic nucleus for 19.5% of patients. Down-regulation of functional connectivity (up to 12%) at these prioritized targets optimally maximized the therapeutic effects. Notably, the priority rank of the subthalamic nucleus significantly correlated with motor symptom severity (Unified Parkinson’s Disease Rating Scale III) in the local cohort. These findings underscore the potential of neural network modeling for advancing personalized brain stimulation therapy, and warrant future experimental investigation to validate its clinical utility.

## Introduction

Focal brain stimulation *via* optical, sonic, electrical, and magnetic means enables adjustable and selectable modulation of network-level activity, thereby eliciting varying therapeutic effects. As one of the most successful clinical interventions, deep brain stimulation (DBS) has demonstrated remarkable symptomatic amelioration in a wide range of neurological [1, 2] and psychiatric conditions [3, 4]. However, the clinical efficacy of brain stimulation is unpredictable on a case-by-case basis, which may be partially due to inter-individual differences in stimulation-induced effects [5, 6], and our incomplete understanding of their neural circuit-level mechanisms [2, 7–10]. To date, there are no theoretical principles or pre-surgical consensus for the determination of desirable stimulation targets (e.g. pallidal *versus* subthalamic DBS) [5, 11–14] and the fine-tuning of stimulation parameters (e.g., current amplitude, frequency, and pulse-width) [8, 15, 16] to optimize a single patient’s outcome.

The functional brain connectome constructed from resting-state functional connectivity is powerful in characterizing the nature of topological organization [17–21] and the neuropathology of the diseased brain [22–25]. Although the majority of fMRI studies have used a case-control approach to detect group effects and this is not directly applicable to clinical situations, a growing body of research has demonstrated that an individual brain can be differentially characterized by its connectome in both healthy [26–28] and disease conditions [29, 30]. This has enabled the development of a variety of whole-brain computational models with an emphasis on clinical applications [6, 17, 23, 24, 31–33].

**Fig. 1:**
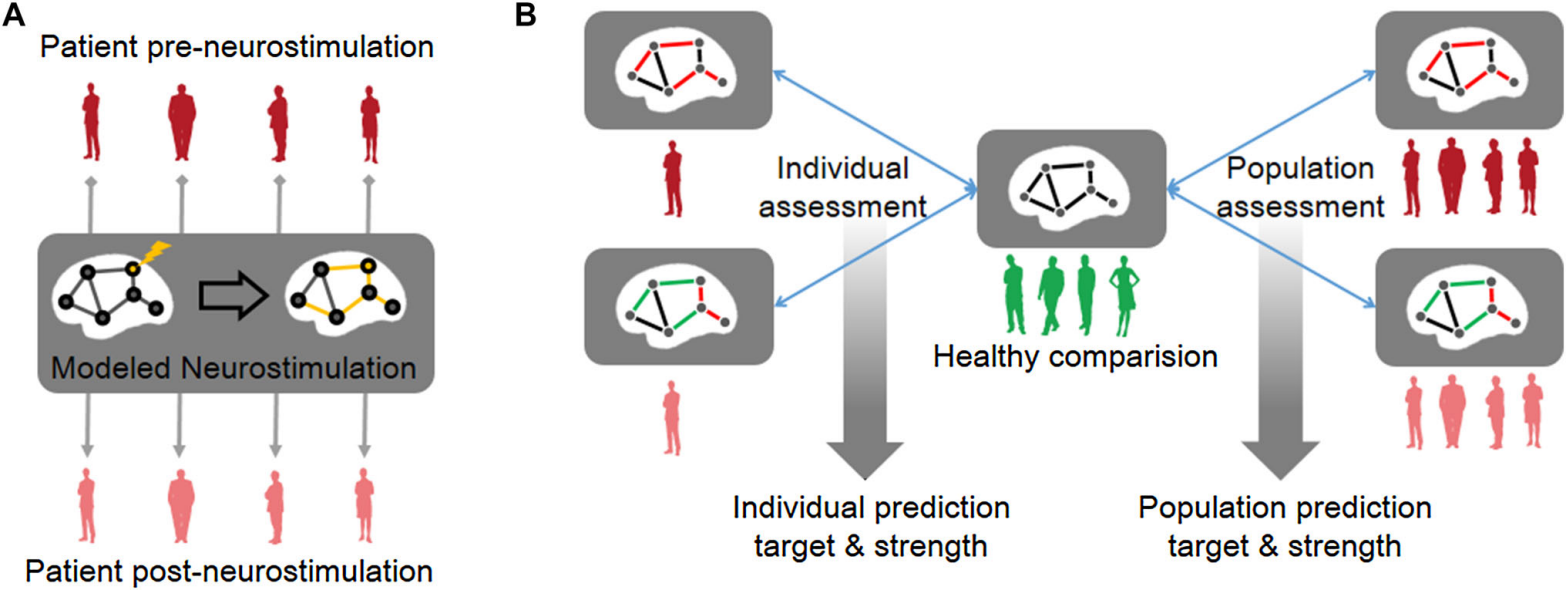
Schematic of whole-brain neurostimulation modeling at the population and individual levels. **A** The pre-neurostimulation functional brain connectome of an individual PD patient (red) is subjected to focal stimulation of a selected target site (yellow node), where neurostimulation generates globally distributed network effects to the whole brain (yellow edges), and then results in a post-neurostimulation connectome (pink). **B** Compared to the healthy connectome (middle, green), abnormal network topology in a single patient (left) or patient population (right) may be partially normalized by neurostimulation. The neurostimulation-induced topological re-balancing effects can be assessed on both population-averaged and individual connectomes.

Emerging evidence has shown that both invasive and noninvasive neurostimulation can reconfigure brain networks and normalize maladaptive functional brain networks along with symptomatic improvement [34–36]. In other words, the therapeutic effects of neurostimulation are attributable to the rectification or rebalancing of abnormal brain-wide network topology towards a healthy regime [7, 37–39]. Following this intuition at the brain-wide scale, we proposed a functional connectome-based neuromodeling approach by which we are able to simulate distributed network effects for different targets and strengths at both the population and individual levels (Fig. 1). For comparison to a group of 46 healthy individuals, we launched a proof-of-principle test in two patient groups: 43 patients with Parkinson’s disease (PD) recruited from the local community and 90 PD patients from a public dataset (as an independent validation). Using fully cross-validated analysis, we identified brain areas mainly located in the basal-ganglia circuits, including the globus pallidus (GP) and subthalamic nucleus (STN), as prime targets in 86.1% of the local and 87.8% of the public cohort. We further determined the optimal range of neurostimulation strength for each targeted area. This computational study allows exploration of the therapeutic potential for a wide array of regions beyond basal ganglia circuits, and the guidance of fine-tuning stimulation parameters in individual patients with PD, which can be integrated as part of a pre-surgical treatment plan in future trials.

## Materials and Methods

### Participants

The locally-enrolled sample consisted of 94 participants, among whom 47 were recruited at the Department of Neurology at Ruijin Hospital with a diagnosis of PD according to the UK Brain Bank criteria. All PD patients received medication and the severity of their motor symptoms was evaluated by study-site personnel and qualified neurologists using the Unified Parkinson’s Disease Rating Scale (UPDRS-III) and the Hoehn-Yahr Staging Scale. Forty-seven healthy comparison (HC) participants were recruited by advertisement and administered the Mini-International Neuropsychiatric Interview (MINI 6.0.0). None of the recruited participants had any neurological or psychiatric conditions, a history of substance abuse, brain injury, or other notable abnormalities upon MRI examination. Four PD patients and one healthy individual were excluded due to severe head motion during MRI scanning (> 2 mm translation or 2.0° rotation). This study was approved by the Review Board at Ruijin Hospital of Shanghai Jiao Tong University and by the Biomedical Research Ethics Committee, Shanghai Institutes for Biological Sciences, Chinese Academy of Sciences. Informed consent was obtained from each participant or their legal guardian upon receiving a complete description of the study. Demographic and clinical characteristics of the 179 participants are summarized in Table 1.

**Table 1:** Demographic and clinical characteristics of local PD patients, public PD patients, and healthy comparison (HC) participants.

	Local PD (*n* = 43)	Public PD (*n* = 90)	HC (*n* = 46)
Age (years)	62.00 ± 6.04	61.70 ± 10.33	31.37 ± 8.30
Gender (No. of males/females)	21/22	61/29	27/19
Duration of illness (years)	4.41 ± 2.54	2.24 ± 1.04	–
UPDRS-III	21.93 ± 11.39	–	–
MDS-UPDRS-III	–	21.28 ± 11.00	–
H-Y scale	1.62 ± 0.53	1.73 ± 0.47	–

Values are mean ± SD; UPDRS-III, Unified Parkinson’s Disease Rating Scale part III; MDS, Movement Disorder Society; H–Y scale, Hoehn and Yahr scale.

Data for an additional set of 93 PD patients with resting-state fMRI were obtained from the Parkinson’s Progression Markers Initiative (PPMI) database. Three cases were excluded due to severe head motion using the same exclusion criteria. All data were fully anonymized as required by HIPAA regulations and participating sites received local Institutional Review Board approval for acquisition of the contributed data. Detailed information about the PPMI patients is available at www.ppmi-info.org.

### MRI Datasets

All local participants were scanned with a standard 12-channel head coil on a Siemens Tim Trio 3.0 T scanner (Erlangen, Germany) at the Institute of Neuroscience, Chinese Academy of Sciences. Each participant was instructed to lie supine in the scanner wearing earplugs with the head snugly fixed by tight but comfortable foam pads. Real-time electrocardiogram and heart rate were continuously monitored throughout the scan (Erlangen, Germany). High-resolution T1-weighted images were acquired using a 3D magnetization-prepared rapid gradient-echo sequence (TR/TE = 2300/3 ms, TI = 1000 ms, flip angle = 9°, FOV = 256 × 256 mm^2^, voxel size = 1 × 1 × 1 mm^3^, 176 consecutive sagittal slices). Resting-state fMRI images from patients were acquired for 300 volumes using a multiband echo-planar imaging sequence (TR/TE = 2000/30 ms, flip angle = 62°, in-plane resolution = 3 × 3 mm^2^, slice thickness = 3 mm, 56 axial slices, multiband acceleration factor = 2). Note that the local PD patients received their medication at the time of the scan. In the HCs, resting-state fMRI images were acquired for 300 volumes with a single-shot echo-planar imaging sequence (TR/TE = 3000/30 ms, flip angle = 90°, in-plane resolution = 2 × 2 mm^2^, 3 mm slice thickness, 47 axial slices). During the resting-state scan, participants were instructed to lie still with their eyes closed, remain awake, and not think of anything in particular (adherence was confirmed by participants immediately after the scan).

Anatomical and resting-state fMRI data from the public PPMI dataset were acquired on Siemens Tim Trio 3.0 T scanners. Structural images were recorded using a protocol similar to that described above. Resting-state fMRI images were acquired for 210 volumes using an echo-planar imaging sequence (TR/TE = 2400/25 ms, flip angle = 80°, in-plane resolution = 3.3 × 3.3 mm^2^, slice thickness = 3.3 mm, 40 axial slices). Further technical details can be found in the MRI operation manual available at http://www.ppmi-info.org/.

### Network Construction

The fMRI data were minimally preprocessed using statistical parametric mapping (http://www.fil.ion.ucl.ac.uk/spm). The first 10 volumes were discarded for signal equilibrium and the remaining volumes were corrected for temporal differences in slice acquisition and rigid-body head movement. The corrected data were spatially normalized to MNI (Montreal Neurological Institute) space and re-sampled to 3-mm isotropic voxels. After normalization, six motion parameters (three for translation and three for rotation) estimated during the realignment process were regressed out and linear drift was removed. A band-pass filter (0.01–0.08 Hz) was applied to remove low-frequency drift and high-frequency respiratory and cardiac noise. We constructed the whole-brain connectivity network using a custom parcellation scheme based on the standard Automated Anatomical Labeling (AAL) atlas with addition of the STN as defined by the ATAG (*Atlas* of The bAsal Ganglia) subcortical atlas [40] (details of a total of 92 brain regions are listed in Table 2). The time-series of all voxels within each region were extracted and averaged to obtain a mean time-series. The functional connectivity *f*_*ij*_ between regions was represented by calculating the Pearson correlation coefficients between the mean time series of any pair of parcellated regions (*i* and *j*). A 92 × 92 connectivity matrix **F** was generated for each participant and then subjected to Fisher’s Z-transformation for subsequent analysis.

**Table 2:** Brain regions defined in this study.

Index	Abbreviation	Description	Lobe
1/2	PreCG	Precentral gyrus	Frontal
3/4	SFGdor	Superior frontal gyrus, dorsolateral	Frontal
5/6	ORBsup	Superior frontal gyrus, orbital part	Frontal
7/8	MFG	Middle frontal gyrus, lateral part	Frontal
9/10	ORBmid	Middle frontal gyrus, orbital part	Frontal
11/12	IFGoperc	Opercular part of inferior frontal gyrus	Frontal
13/14	IFGtriang	Area triangularis	Frontal
15/16	ORBinf	Orbital part of inferior frontal gyrus	Frontal
17/18	ROL	Rolandic operculum	Frontal
19/20	SMA	Supplementary motor area	Frontal
21/22	OLF	Olfactory cortex	Frontal
23/24	SFGmed	Superior frontal gyrus, medial part	Frontal
25/26	ORBsupmed	Superior frontal gyrus, medial orbital part	Frontal
27/28	REC	Gyrus rectus	Frontal
29/30	INS	Insula	Insular
31/32	ACG	Anterior cingulate gyrus	Limbic
33/34	DCG	Middle cingulate	Limbic
35/36	PCG	Posterior cingulate gyrus	Limbic
37/38	HIP	Hippocampus	Limbic
39/40	PHG	Parahippocampal gyrus	Limbic
41/42	AMYG	Amygdala	Limbic
43/44	CAL	Calcarine sulcus	Occipital
45/46	CUN	Cuneus	Occipital
47/48	LING	Lingual gyrus	Occipital
49/50	SOG	Superior occipital	Occipital
51/52	MOG	Middle occipital	Occipital
53/54	IOG	Inferior occipital	Occipital
55/56	FFG	Fusiform gyrus	Temporal
57/58	PoCG	Postcentral gyrus	Parietal
59/60	SPG	Superior parietal lobule	Parietal
61/62	IPL	Inferior parietal lobule	Parietal
63/64	SMG	Supramarginal gyrus	Parietal
65/66	ANG	Angular gyrus	Parietal
67/68	PCUN	Precuneus	Parietal
69/70	PCL	Paracentral lobule	Parietal
71/72	CAU	Caudate nucleus	Basal ganglia
73/74	PUT	Putamen	Basal ganglia
75/76	PAL	Globus pallidus	Basal ganglia
77/78	STN	Subthalamic nucleus	Basal ganglia
79/80	THA	Thalamus	Basal ganglia
81/82	HES	Transverse temporal gyri	Temporal
83/84	STG	Superior temporal gyrus	Temporal
85/86	TPOsup	Superior temporal pole	Temporal
87/88	MTG	Middle temporal gyrus	Temporal
89/90	TPOmid	Middle temporal pole	Temporal
91/92	ITG	Inferior temporal gyrus	Temporal

### Construction of a 1024-Region Template

We also constructed a brain template with 1024 regions to cross-validate whether our results were biased by the parcellation scheme. In doing so, 1024 seeds were randomly distributed into 92 brain regions of the AAL atlas with a probability proportional to the size of the regional volume. Two additional constraints were set during the distribution of seeds: each region had at least one seed and all seeds were equally allocated between the two hemispheres. This seeding procedure determined how many sub-regions had to be further generated for each original AAL-based brain region. Then, a k-means method was applied to assign the voxels within the original region into k sub-regions based on their spatial locations.

### Whole-Brain Network Model of Neurostimulation

We first simulated the network effects of neurostimulation by considering the constructed connectome **F** as the resultant network of information spread or diffusion over a direct network **D** for individual patients. The rationale here was that the local effect is the action of stimulation imposed on the direct network **D**, and the globally distributed effect is the propagated result of the local effect on the direct network. As such, we used a network deconvolution algorithm [41] to derive a direct network **D** from the observed network **F**, and used the corresponding transformation rule, transitive closure, to simulate the global effects of local stimulation on a large-scale neuronal network (a detailed description of the modeling is provided below). We then denoted a hypothetical neurostimulation event as a regulation operator **P**, which was imposed on the direct network **D** (i.e., ${\mathbf{D}}^{'} = {\mathbf{D}} \odot {\mathbf{P}}$, where ⊙ is the element-wise product of two matrices). If neurostimulation targets one pair of bilateral loci, most **P** entries have a value of one, with the exception of the two columns and two rows that are directly connected to the locus. For simplicity, the same value that represents the neurostimulation strength is applied to the remaining entries in **P**: a value > 1 signifies up-regulation, whereas a value < 1 indicates down-regulation (e.g., 1.5 represents a 50% up-regulation, 0.7 represents 30% down-regulation). Finally, the neurostimulation-tuned direct network ${\mathbf{D^{\prime}}}$ is convolved with transitive closure to obtain the post-neurostimulation connectivity matrix ${\mathbf{F^{\prime}}}$ (Fig. 1A).

**Fig. 2:**
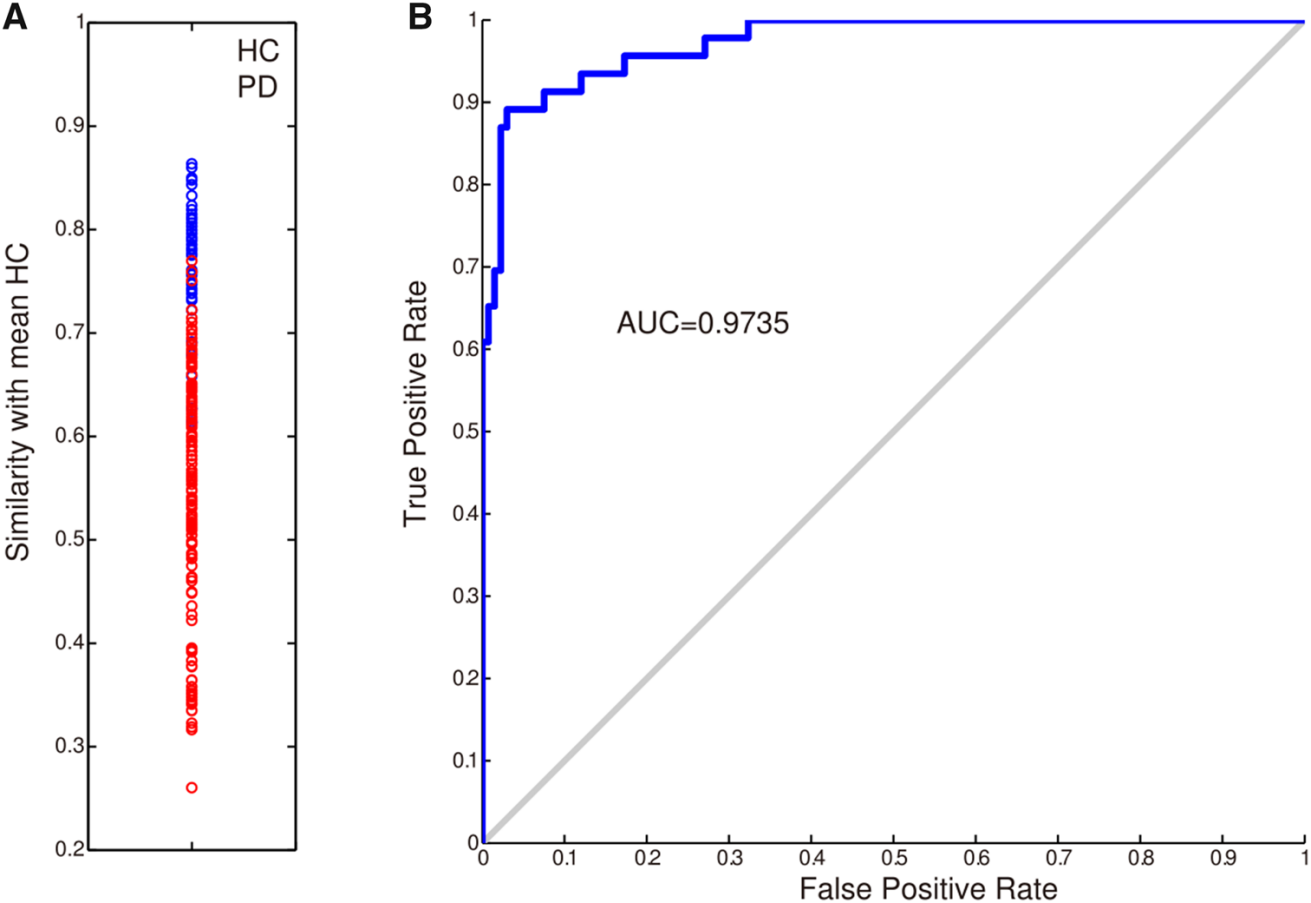
Connectome similarity as a discriminative index between PD patients and HCs. **A** Each individual’s connectome similarity with the group-averaged HC connectome; red dots, PD patients; blue dots, HCs. **B** Receiver-operator characteristic curve of each individual’s connectome similarity in (**A)** to discriminate PD and HC when the discrimination threshold is varied. AUC, area under the curve.

The simulated outcome of neurostimulation was evaluated by quantitatively measuring the similarity between a group-averaged healthy matrix obtained from HCs and an individual or group-averaged post-neurostimulation matrix from PD patients. Connectomic similarity was quantified as the Pearson correlation coefficient between two vectorized (concatenated by rows) upper triangles of the connectivity matrix. This index was appropriate to characterize the discriminant difference between HCs and PD patients, as shown in Fig. 2. In order to compare the outcomes across brain regions, individuals, and groups, we standardized all outcome similarities using *relative change*, defined as follows:

$$ relative\,change = \frac{{CC\left( {poststim, healthy} \right) - CC\left( {prestim,healthy} \right)}}{{CC\left( {prestim, healthy} \right)}} \times 100\%. $$

*Relative change* was used as a quantitative measure to represent the percentage improvement in connectomic similarity towards a healthy regime that was achieved by neurostimulation. The higher the connectomic similarity to the matrix of the HC group, the better was the simulated therapeutic effect. At each targeted brain region, the magnitude of up- or down-regulation that resulted in the highest *relative change* was deemed to be the best strength. All target regions were then ranked according to their *relative change* at the optimal neurostimulation strength. Higher ranks indicated desirable stimulation candidates with better therapeutic effects. All simulations were made at both single patient and patient group levels, as illustrated in Fig. 1B.

### Derivation of a Transformation Rule for Local-Global Network Effects

Although the neurobiological and therapeutic mechanisms underlying a variety of brain stimulation approaches are not fully understood [8, 42–44], the network effects of focal neurostimulation can be roughly categorized into two types: local and distributed brain alterations [1, 44, 45]. The local effect that is generated by direct physical stimulation is believed to elevate excitation or inhibition in the target area [4, 8]. The distributed network effect, considered a global change, is assumed to account for all reverberating effects of the target loci and other connected regions. The challenge of computational modeling of stimulation lies in the lack of effective rules to estimate complex distributed network effects at the whole-brain scale [1, 6, 17, 23, 45, 46]. Given the existence of neuronal dynamics and parallel circuits (direct and indirect pathways), we hypothesized that the functional connectivity matrix **F** measured for individuals in the spontaneous state intrinsically contains a myriad of direct and indirect interactions among distant brain regions. As network size increases, particularly in whole-brain computational modeling, a considerable amount of indirect edges (connections between regions) may reflect second-, third- or even higher-order interactions between nodes (regions). It is therefore expected that the functional connectivity matrix **F** constructed for each individual in the spontaneous state contains direct and additional indirect relationships among distant regions, particularly considering the existence of neuronal dynamics and parallel circuits (pathways) [47]. Hypothetically, the functional brain connectome **F** is a resultant network of information spread or diffusion from a direct network **D**. Therefore, a network deconvolution algorithm was originally proposed to identify direct effects and weights from an observed correlation matrix in which both direct and indirect dependencies exist [41]. The corresponding transform, referred to as transitive closure, is the reverse process which derives the indirect effects from direct effects [41]. Two transforms were used to model the local-global network effect transformation of neurostimulation.

Provided that indirect effects embedded in the brain circuitry are hypothetically composed of an infinite number of linear parts (2nd, 3rd, ……), the functional connectome **F** can be mathematically described as follows:

$$ \underbrace {{\mathbf{F}}}_{\text{original}} = \underbrace {{\mathbf{D}}}_{\text{direct}} + \underbrace {{{\mathbf{D}}^{2} + {\mathbf{D}}^{3} + \cdots }}_{\text{indirect}} $$

Since **F** is intrinsically a symmetrical and positive definite, an analytical solution of direct network **D** is in the form of:

$$ {\mathbf{D}} = {\mathbf{UST}}^{\top} , $$

where ${\mathbf{U}}^{\top} {\mathbf{U}} = {\mathbf{I}}$; the columns of **U** are the orthonormal eigenvectors of **F**, and **S** is a diagonal matrix composed by a linear-fractional transformation of the eigenvalues ($\uplambda_{i}$) of matrix **F**:

$$ {\mathbf{S}} = diag\left( {\frac{{\uplambda_{1} }}{{1 +\uplambda_{1} }},\frac{{\uplambda_{2} }}{{1 +\uplambda_{2} }}, \ldots \frac{{\uplambda_{n} }}{{{1 + \lambda }_{n} }}} \right). $$

Note that the direct network **D** is not sparse, such that it contains all core neural circuits or pathways and the corresponding probabilistic weights of individual edges representing direct interactions.

Inversely, given a direct network **D**, the original network **F** can also be computed analytically:

$$ {\mathbf{F = V}}\sum {\mathbf{V}}^{\top} , $$

where ${\mathbf{V}}^{\top} {\mathbf{V}} = {\mathbf{I}}$; the columns of **V** are the orthonormal eigenvectors of **D**, and Σ is a diagonal matrix composed by a linear-fractional transformation of the eigenvalues ($\upeta_{i}$) of matrix **D**:

$$ \sum { = diag\left( {\frac{{\eta_{1} }}{{1 - \eta_{1} }},\frac{{\eta_{2} }}{{1 - \eta_{2} }}, \ldots \frac{{\eta_{n} }}{{1 - \eta_{n} }}} \right)} . $$

Evidently, transitive closure (TC) and network deconvolution (ND) are inverse operators, i.e., ${\mathbf{F}} = TC  { \left( {ND \left( {\mathbf{F}} \right)} \right)}$ and ${\mathbf{D}} = ND \left( {TC \left( {\mathbf{D}} \right)} \right)$. The potential benefits of this analytically reversible procedure are many-fold. First, key driving sources of a measured connectome and their associated weights can be deduced (i.e., relative contributions). Second, the location and strength of focal manipulation applied to the direct network can be assessed to predict whether or not a desired network can be achieved. Third, the transformation rule is empirically derived from the functional connectivity matrix recorded from each individual, which is not theoretically hypothesized and uniformly applied to a group of individuals. Last, it has enabled the study of network functions and properties in the core domain of direct interactions, rather than in the originally measured network plagued by complicated indirect interactions. Investigation in this direct network may help to unveil fundamental topological and dynamic properties of a network, and to improve prediction performance (e.g., the area under the precision-recall curve and the area under the receiver operating characteristic curve) [41]. In short, network deconvolution provides a succinct analytical solution to estimate a participant-specific, local-global transformation rule of information flow propagating over a network, which may approximately account for complex processes over the whole brain triggered by focal stimulation.

It is critically important to note that the absolute value of the largest eigenvalue of the matrix should be < 1 before subjecting it to network deconvolution or transitive closure to ensure the convergence of both procedures. Although the original functional connectivity matrix that was constructed from resting fMRI data fulfills this criterion, the matrix after neurostimulation (i.e., increasing the connectivity strength by 60%) may not obey this constraint. Thus, a scaling factor α was used to multiply the matrix to rectify this issue, as defined in the expression below [41]:

$$ \upalpha \le \hbox{min} \left( {\frac{\upbeta}{{\left( {1 -\upbeta} \right)\uplambda_{ + } }},\frac{{ -\upbeta}}{{\left( {1 +\upbeta} \right)\uplambda_{ - } }}} \right), $$

where λ_+_ and λ_-_ are the largest positive and smallest negative eigenvalues of the connectivity matrix. β is an empirically determined positive parameter which should be < 1, where the absolute eigenvalue of the direct matrix is supposed to be < β. Considering a wide range of neurostimulation strengths that might be necessary for a heterogeneous patient population, β was set to 0.5, similarly determined in previous work [41].

We first calculated the upper bound of the scaling factor for the connectivity matrices from patients and HCs and determined the largest α value that satisfied the upper inequality expression for each matrix:

$$ \upalpha = \hbox{min} \left( {\upalpha^{1} ,\upalpha^{2} , \ldots ,\upalpha^{i} , \ldots ,\upalpha^{n} } \right), $$

$$ \upalpha^{i} = \hbox{min} \left( {\frac{\upbeta}{{\left( {1 -\upbeta} \right)\uplambda_{ + }^{i} }},\frac{{ -\upbeta}}{{\left( {1 +\upbeta} \right)\uplambda_{ - }^{i} }}} \right). $$

Here, for the *i*th network matrix of total *n* matrices, α^i^ is the upper bound determined by the largest positive eigenvalue ($\uplambda_{ + }^{i}$) and smallest negative eigenvalue ($\uplambda_{ - }^{i}$) of this matrix. Finally, all the matrices were multiplied by the determined constant α and the linearly scaled matrices were used for neurostimulation.

### Study Design, Statistical Analysis, and Cross-Validation

As a proof-of-principle study, we rigorously tested the robustness and consistency of the simulated results using various cross-validation procedures. To validate whether the model is biased by the inclusion or exclusion of participant data, we randomly sampled half of the PD and HC groups 1000 times. Unblinding of between-group labels is also necessary to generate cross-validated simulation robustness. We randomly sampled half of the HC group 1000 times as the “patient group” to validate whether the present strategy for target is biased by the modeling per se. Moreover, we used the 1024-region parcellation template (described above) to determine whether the result of targets or strengths was biased by a specific brain parcellation scheme. For the results of individual patients, we assessed the statistical significance (one-sample *t* test) and the effect size (Cohen’s *d* value) of the simulated therapeutic effects for all brain regions. We assigned a rank for each region across individuals and summarized the number of occurrences of the ranks. The optimal neurostimulation strength of each region for each patient was also plotted and summarized statistically (one-sample *t*-test). For external validation, we repeated the same procedure using an independent public dataset (PPMI PD patients).

To reveal the extent of rectification of network topology achieved through neurostimulation, we conducted edge-wise statistical comparison between the two groups. The results of two-sample *t*-tests after Bonferroni correction for multiple comparisons (*P* < 0.05 corrected) were categorized into three types: removed abnormal connections (i.e., significant difference between the PD and HC groups before, but not after neurostimulation), newly-emerged abnormal connections, and unchanged abnormal connections. Finally, to examine the relationship between the simulated priority of regions and symptom severity, a Kendall rank correlation was calculated between each target’s rank and the UPDRS-III score for the two datasets.

## Results

### Internal Validation

**Fig. 3:**
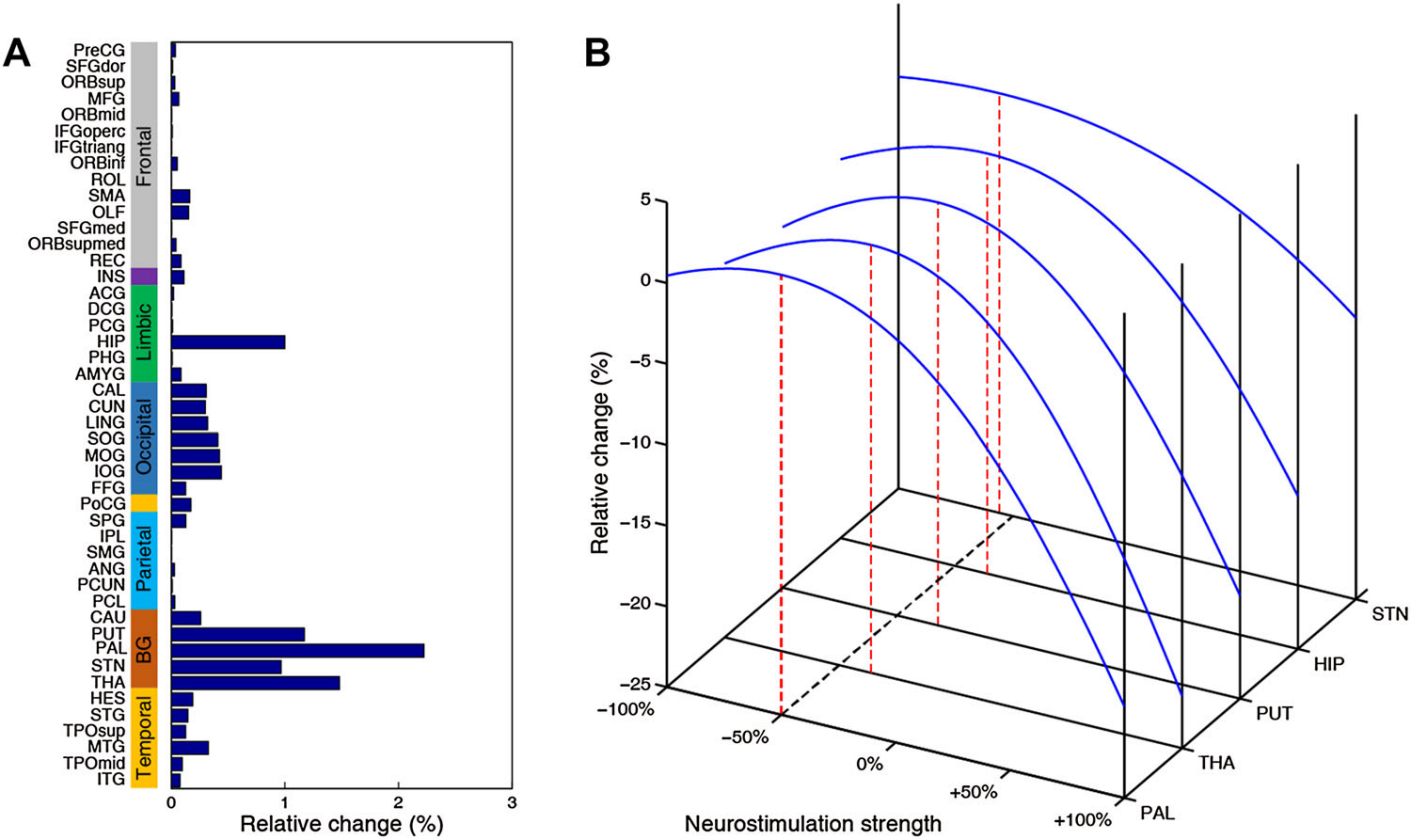
Neurostimulation target identification for the local PD group. **A** The highest relative changes of all brain regions after neurostimulation at the population level plotted for the local cohort. The parcellated regions are shown on the Y-axis and categorized into frontal, insular, limbic, occipital, parietal, and temporal lobes, and the basal ganglia. **B** Relative change plotted against different neurostimulation strengths for each top-five target. Red bars, optimal neurostimulation strength with the largest relative change at each region.

**Fig. 4:**
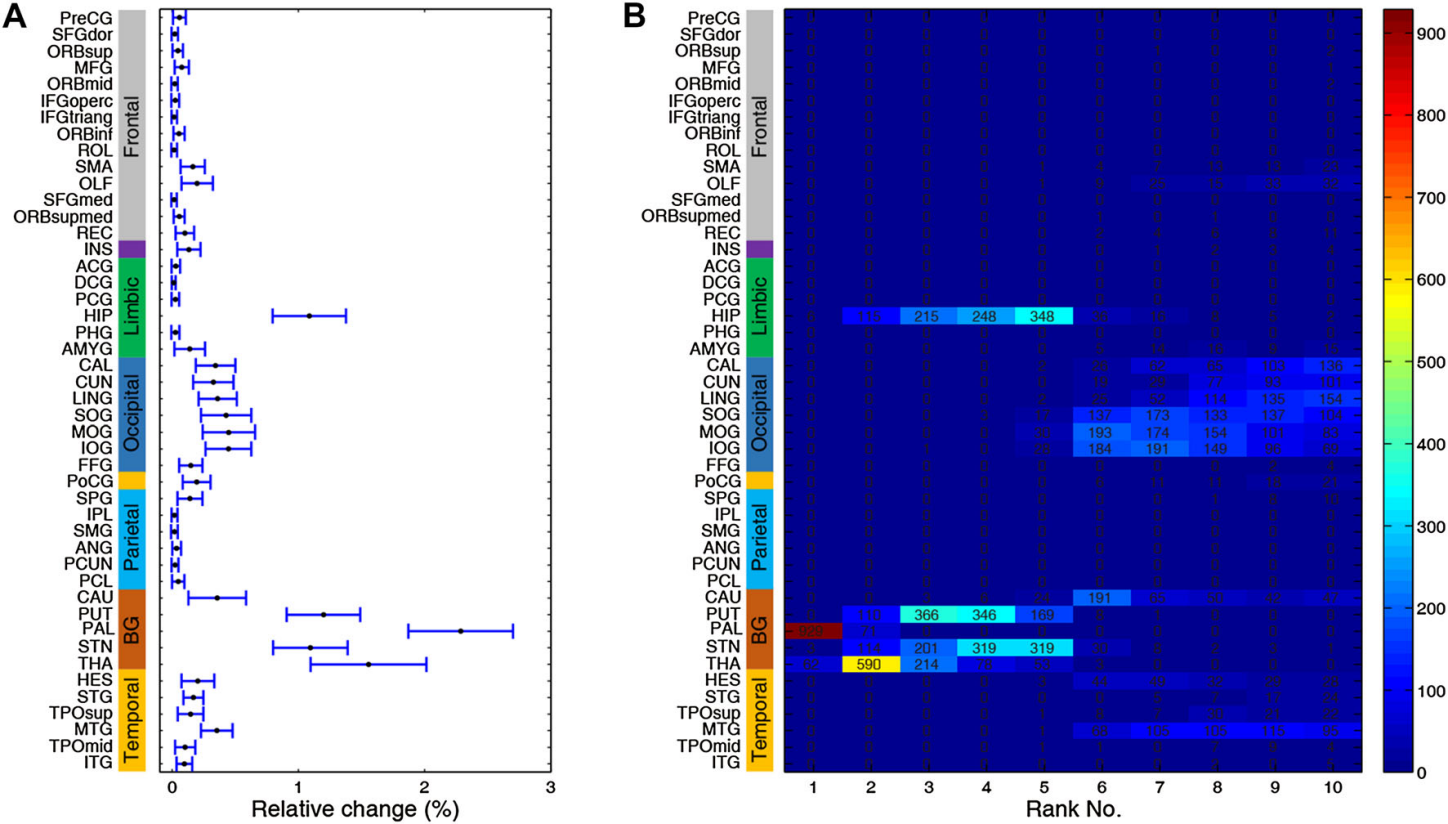
Group sub-sampling cross-validation. An iterative leave-half-out cross-validation procedure (1000 iterations) was applied to local PD and HCs. **A** Mean relative change of 1000 repetitions for each region (error bars, standard deviation). **B** Total occurrence times of top-10 regions throughout 1000 rounds of simulation, in which the GP was identified as the best candidate target 967 times.

**Fig. 5:**
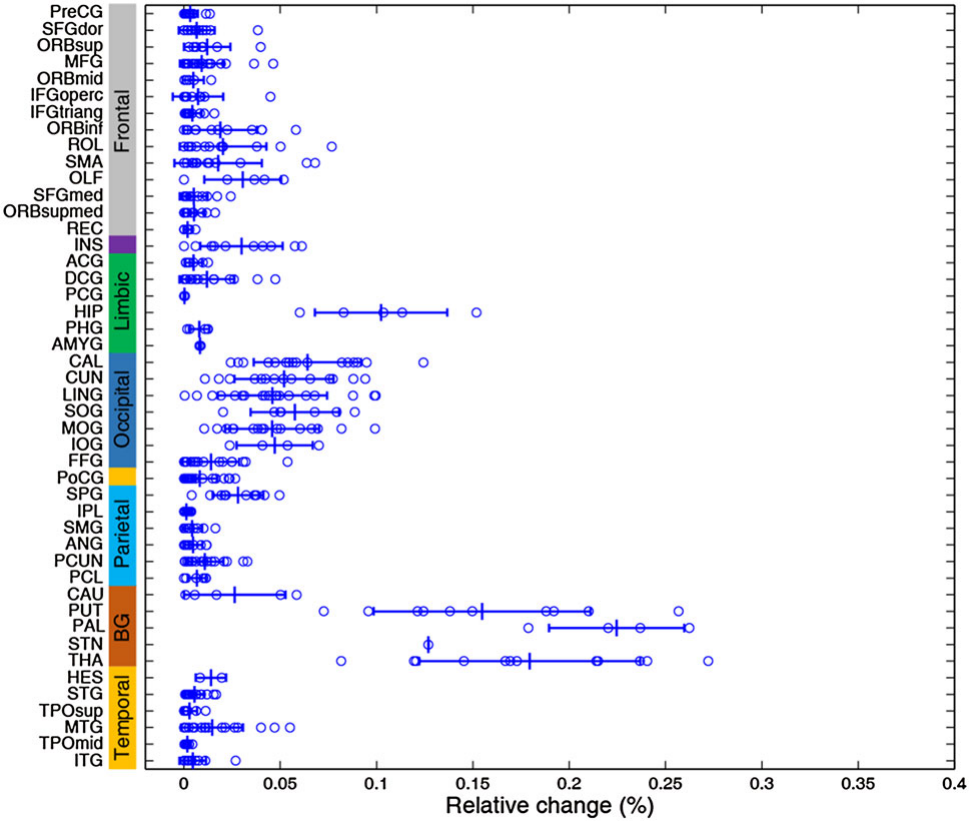
Validation of the brain parcellation scheme. The original parcellation was subdivided into a new 1024 parcellation scheme to construct a high-resolution functional connectome for the identification of neurostimulation targets. The relative change of each subdivided area is plotted (open circles) along the original parcellation together with its mean (vertical line) and standard deviation (error bars).

**Fig. 6:**
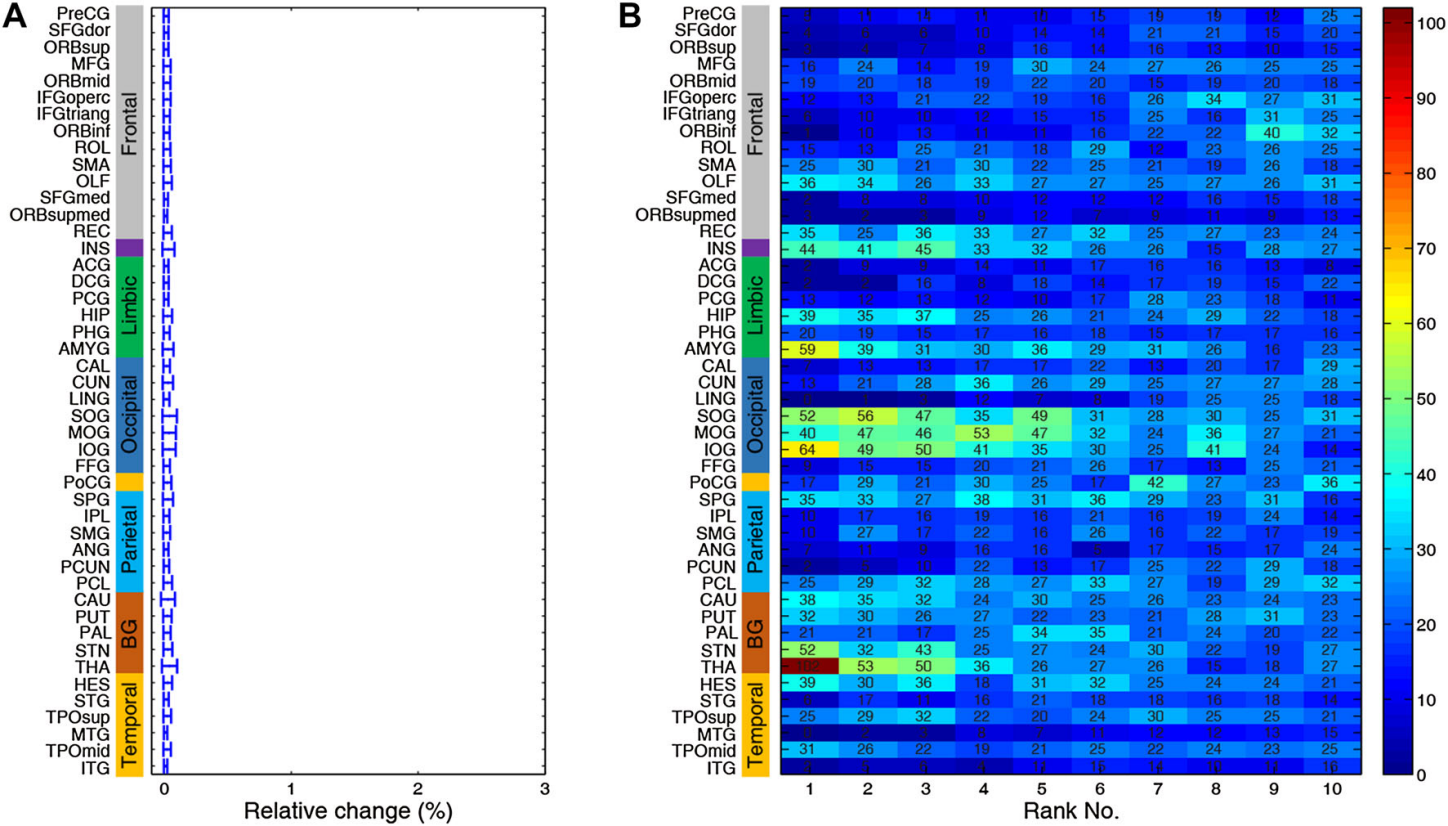
Cross-validation with unblinded between-group labels. We randomly sampled half of the 46 HCs as one “patient group” for target identification 1000 times. **A** Mean relative change of 1000 iterations for each region (error bars, standard deviation). All regions show extremely low relative changes. **B** Total occurrence times of the top-10 regions throughout 1000 tests, demonstrating that nearly all regions had a random chance of being a candidate target.

**Fig. 7:**
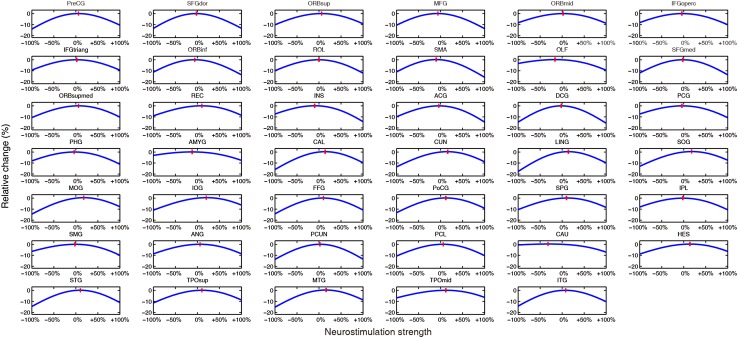
Strength identification at different brain sites for the local PD group. Relative change is plotted against different neurostimulation strengths within each region. The simulated results of the remaining 41 brain regions (complementary to Fig. 2B) are shown here. Red bars, optimal modulation strength with the largest relative change in each region.

We began by screening all 46 bilateral brain regions as potential targets in the local PD group. The most effective neurostimulation strength for each region (resulting in the largest percentage improvement) was used to compare outcomes among all regions. At the group level, the GP was clearly identified as the best choice for neurostimulation, exhibiting the largest relative change of 2.22% (Fig. 3A). This indicates that the greatest improvement in the local PD group may be achieved by focal modulation of the GP. The STN, thalamus, and putamen evidently emerged as top-ranked targets with relative changes of 0.97%, 1.48%, and 1.17%, respectively. The hippocampus was a desirable target for which neurostimulation attained a 1.00% improvement. Importantly, these results withstood a 1000-round sub-sampling cross-validation process (Fig. 4). Using a delicate 1024-region parcellation template to repeat the analysis, we confirmed that the GP, STN, putamen, thalamus, and hippocampus remained top-ranked candidates for neurostimulation (Fig. 5). Note that the performance of different subregions within the thalamus varied widely, suggesting that specific nuclei of the thalamus may be extremely sensitive to stimulation. Furthermore, we randomly selected half of the HC sample as “patients” for target identification to demonstrate that this model would not biasedly choose any specific region as a potential target (Fig. 6). Meanwhile, neurostimulation strength, another key element of a typical stimulation protocol, was estimated for each candidate target. In the local PD group, optimal neurostimulation strengths for the top five targets (GP, STN, putamen, thalamus, and hippocampus) were determined to be 50%, 56%, 36%, 32%, and 32% down-regulation of their original connectivity strengths, respectively (Fig. 3B). Neurostimulation strengths for the remaining regions are displayed in Fig. 7.

**Fig. 8:**
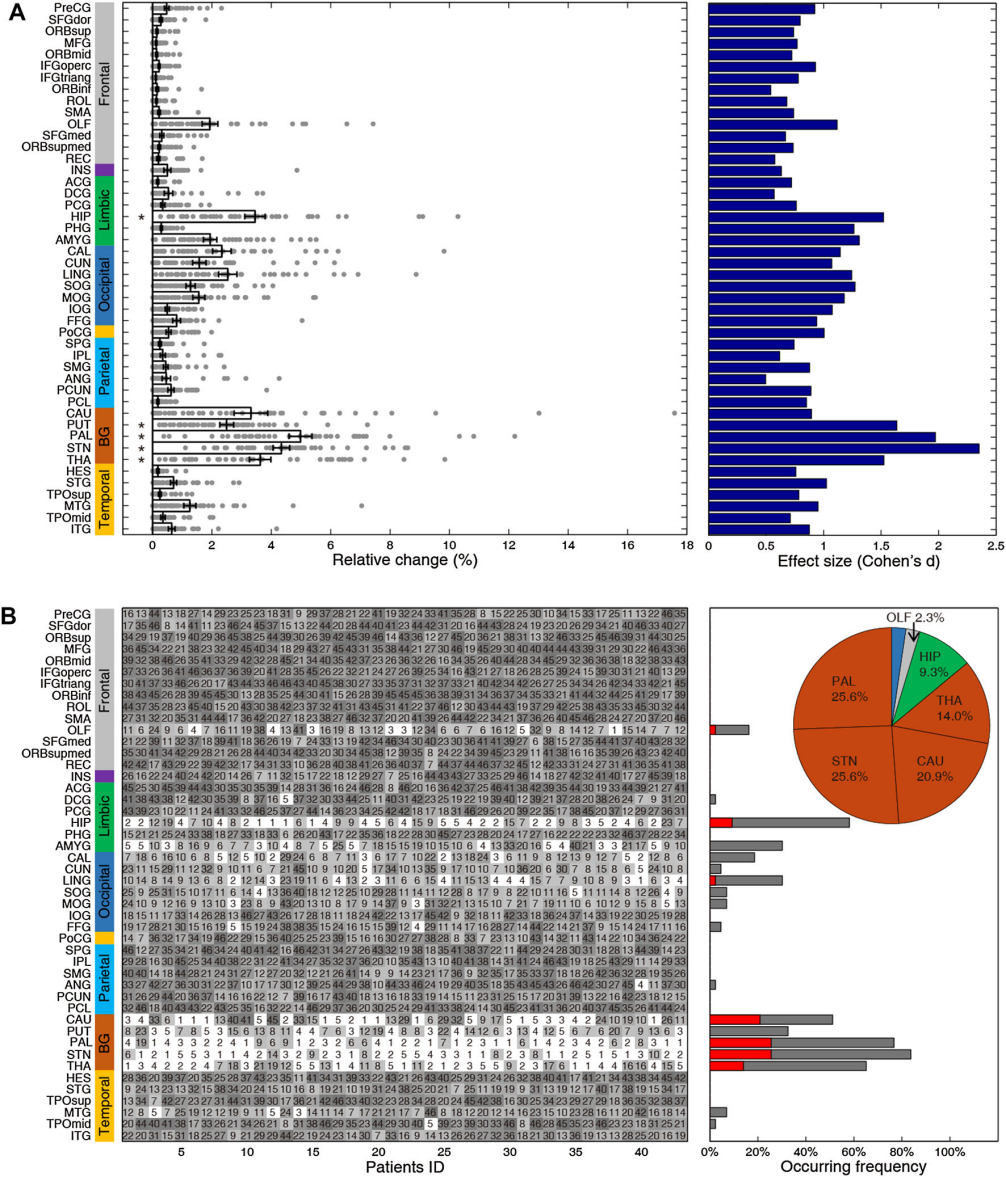
Neurostimulation target identification in individual local PD patients. **A** Left panel, relative changes (x-axis) of all brain regions for each patient (dots) (bar plot with error bars represent mean ± SEM; *P < 10^−11^ for top-five targets, one-sample *t*-test). Right panel, corresponding effect sizes (Cohen’s *d* value) for each region. **B** Left panel, complete rank of all regions for each patient; x-axis, patient ID in the local PD cohort. The top-five brain regions of each patient are highlighted in white. Right panel, occurrence frequencies of the best (red) and top-five (grey) sites. The pie plot summarizes the occurrence frequency of the best target for each patient.

**Fig. 9:**
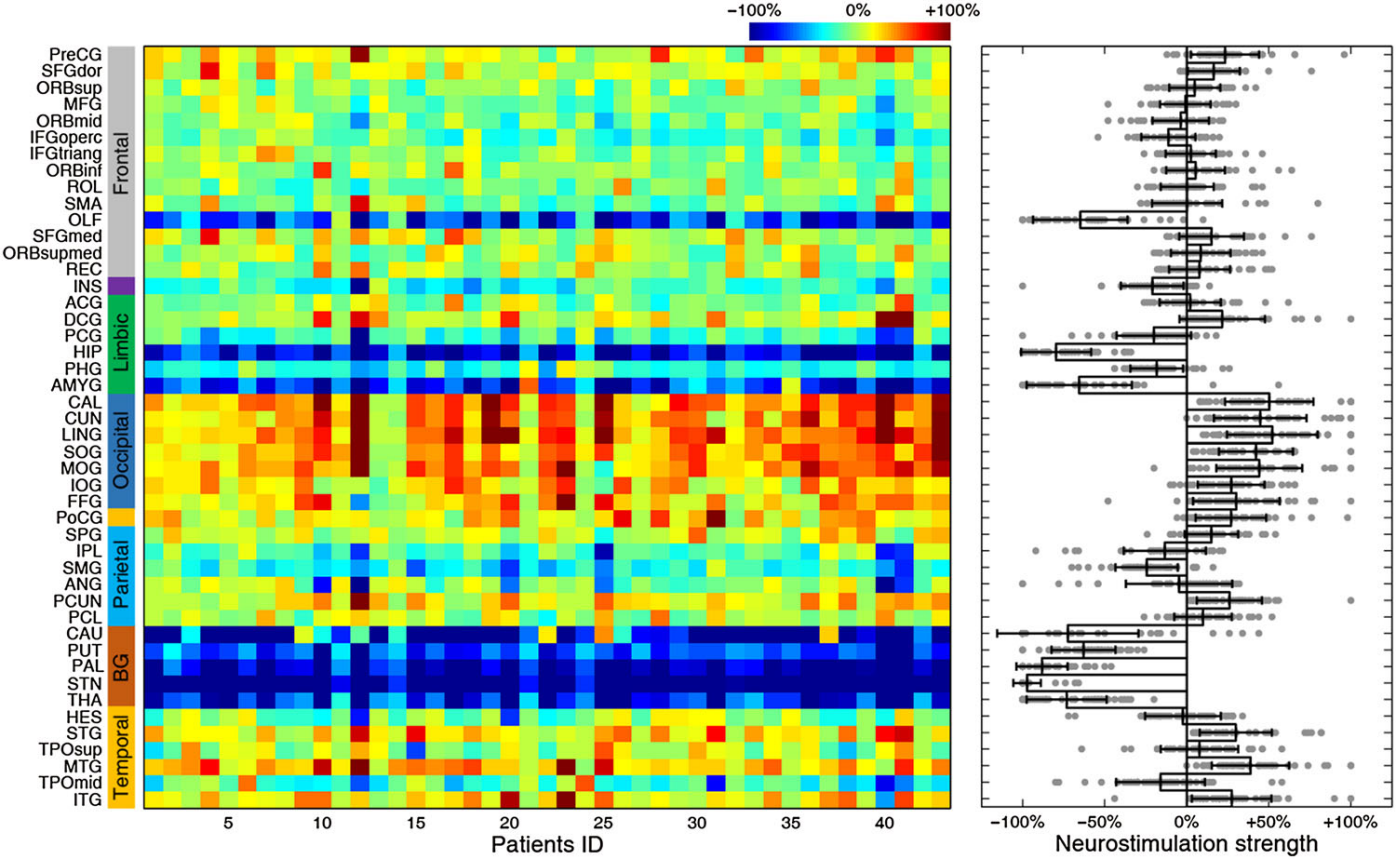
Neurostimulation strength identification in individual local PD patients. Left panel, optimal neurostimulation strengths for all regions for each patient (color bar, range of simulated neurostimulation strengths). Right panel, neurostimulation strengths (x-axis) of all regions in all patients (dots), where the magnitudes of basal ganglia and hippocampus areas were significantly different from other regions (*P* < 10^−11^) (bar plot with error bars represents mean ± SEM).

We then moved on to target and strength identification for individual patients. For each brain region, we plotted the relative change in individual patients (Fig. 8A, left panel). In the local cohort, the top-five regions (GP, STN, thalamus, putamen, and hippocampus) consistently demonstrated significant improvement (Fig. 8A, left panel), with rather large effect sizes (Fig. 8A, right panel). We subsequently plotted the complete rank of all regions as potential candidates for each patient in the local cohort (Fig. 8B, left panel) and counted their occurrence frequencies as the best or top-five choice (Fig. 8B, right panel). Evidently, the GP was the best choice for 11 (25.6%) and a top-five choice for 33 (76.7%) patients, and the STN was the best choice for 11 (25.6%) and a top-five choice for 36 (83.7%) patients (pie plot in Fig. 8B, right panel). Meanwhile, the caudate, thalamus and hippocampus were the best targets for 9 (20.9%), 6 (14.0%), and 4 (9.3%) patients, and a top-five choice for 22 (51.2%), 28 (65.1%), and 25 (58.1%) patients, respectively. In sum, the nuclei of the basal ganglia circuit were identified as the best choices for 86.1% and as a top-five choice for 97.7% of the local cohort, in striking agreement with previous clinical results [48]. Intriguingly, we found a significant relationship between the rank of the STN in this cohort and the severity of symptoms as indexed by the UPDRS-III (*P* < 0.05, Kendall rank correlation), but no other top sites. Moreover, we performed personalized identification of optimal strengths for each region in individual patients of the local cohort (Fig. 9, left panel). Regardless of the marked variability between individuals, consistent down-regulation (Fig. 9, right panel) of basal ganglia- and hippocampus-related functional connectivity substantially contributed to the rectification of dysfunctional networks.

### External Validation

**Fig. 10:**
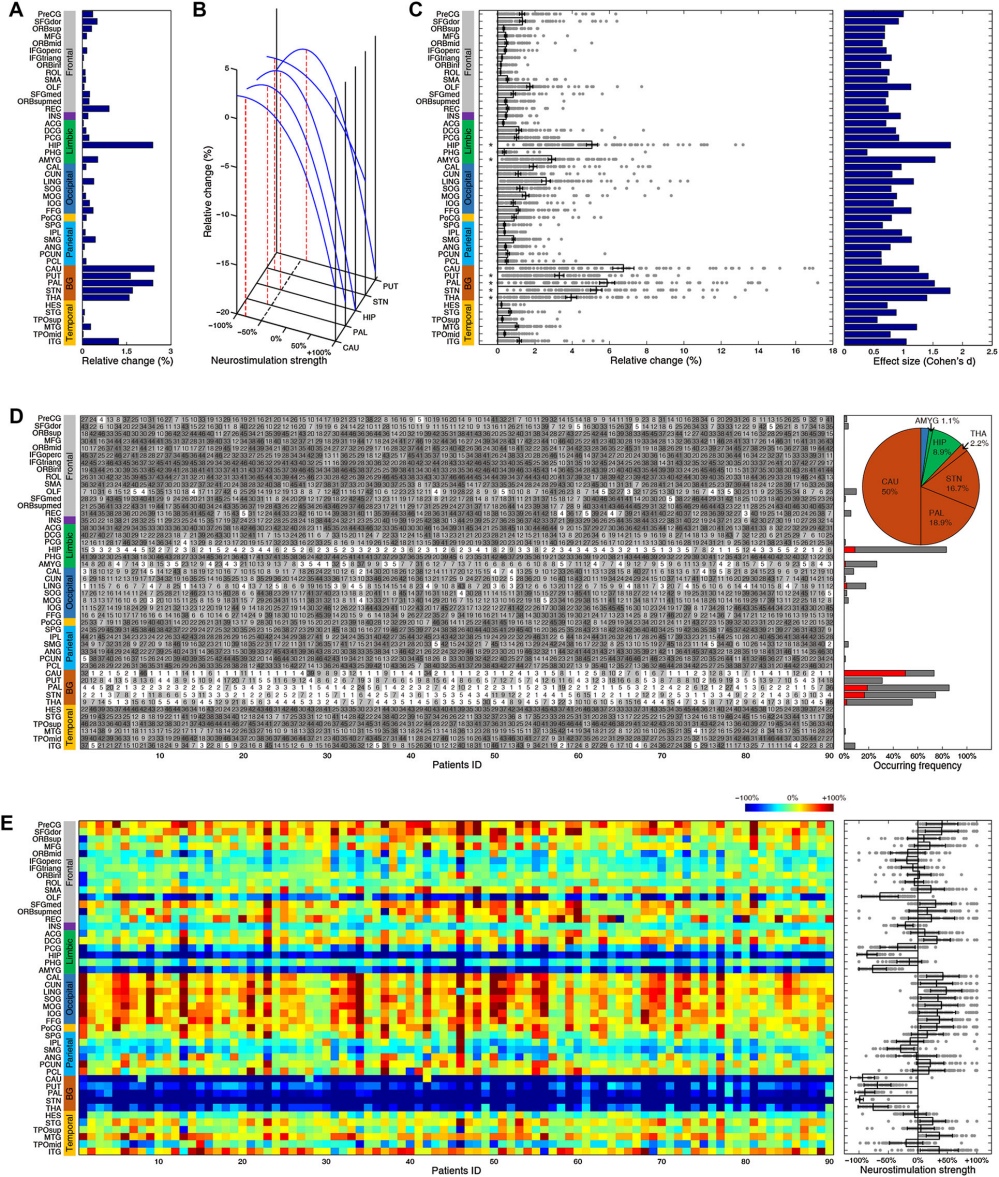
Identification of neurostimulation targets and strengths in the public PD cohort. **A**, **B** The results for targets **(A)** and strengths **(B),** as in Fig. 2. **C** Left panel, relative changes (x-axis) of all brain regions for each patient (dots) (bar plot with error bars represent mean ± SEM; *P < 10^−20^ for top-five targets, one-sample t-test). Right panel, corresponding effect sizes (Cohen’s *d* value) for each region. **D** Left panel, complete rank of all regions for each patient; x-axis, patient ID in the local PD cohort. The top-five brain regions of each patient are highlighted in white. Right panel, occurrence frequencies of the best (red) and top-five (grey) sites. The pie plot summarizes the occurrence frequency of the best target for each patient. **E** Neurostimulation strength identification in individual public PD patients. Left panel, optimal neurostimulation strengths for all regions for each patient (color bar, range of simulated neurostimulation strengths). Right panel, neurostimulation strengths (x-axis) of all regions in all patients (dots) (bar plot with error bars represents mean ± SEM).

As a robust test of generalizability, we applied the present modeling analysis to a completely independent validation dataset from the public PPMI database, which provides resting-state fMRI data for 90 patients with PD (Fig. 10). We found that nuclei of the basal ganglia circuit and hippocampus stood out as prioritized selections, in agreement with results from the local PD cohort (Fig. 10A). Correspondingly, we found that the optimal neurostimulation strengths for the GP, STN, caudate, putamen, and hippocampus were almost identical to those in the local group (58%, 82%, 84%, 40%, and 52% down-regulation, respectively; Fig. 10B). At the single patient level, the top-five targets selected for each patient in the public cohort were also consistent with those of the local group. Stimulation of the GP, STN, thalamus, putamen, hippocampus, and amygdala showed significant percentages of improvement (Fig. 10C, left panel) in this group with large effect sizes (Cohen’s *d* > 1.3, Fig. 10C, right panel). The GP was the best target for 17 (18.9%) and a top-five choice for 77 (85.6%) patients, and the STN was the best target for 15 (16.7%), and a top-five choice for 67 (74.4%) patients (Fig. 10D). The caudate, thalamus, hippocampus, and amygdala were the best choices for 45 (50.0%), 2 (2.2%), 8 (8.9%), and 1 (1.1%) individual patients, and a top-five choice for 66 (73.3%), 50 (55.6%), 75 (83.3%), and 24 (26.7%) patients (Fig. 10D), respectively. We therefore summarize the nuclei of the basal ganglia circuits as prioritized targets for 87.8% and as top-five choices for 100% of this public group. Note that we did not find a significant relationship between the ranks of any top-five sites and the severity of motor symptoms in the public cohort (*P *> 0.05, Kendall rank correlation). Meanwhile, personalized identifications of the optimal strength for each region are shown for the public cohort in Fig. 10E; they manifested consistent down-regulation.

### Reconfiguration of Network Topology by Neurostimulation

**Fig. 11:**
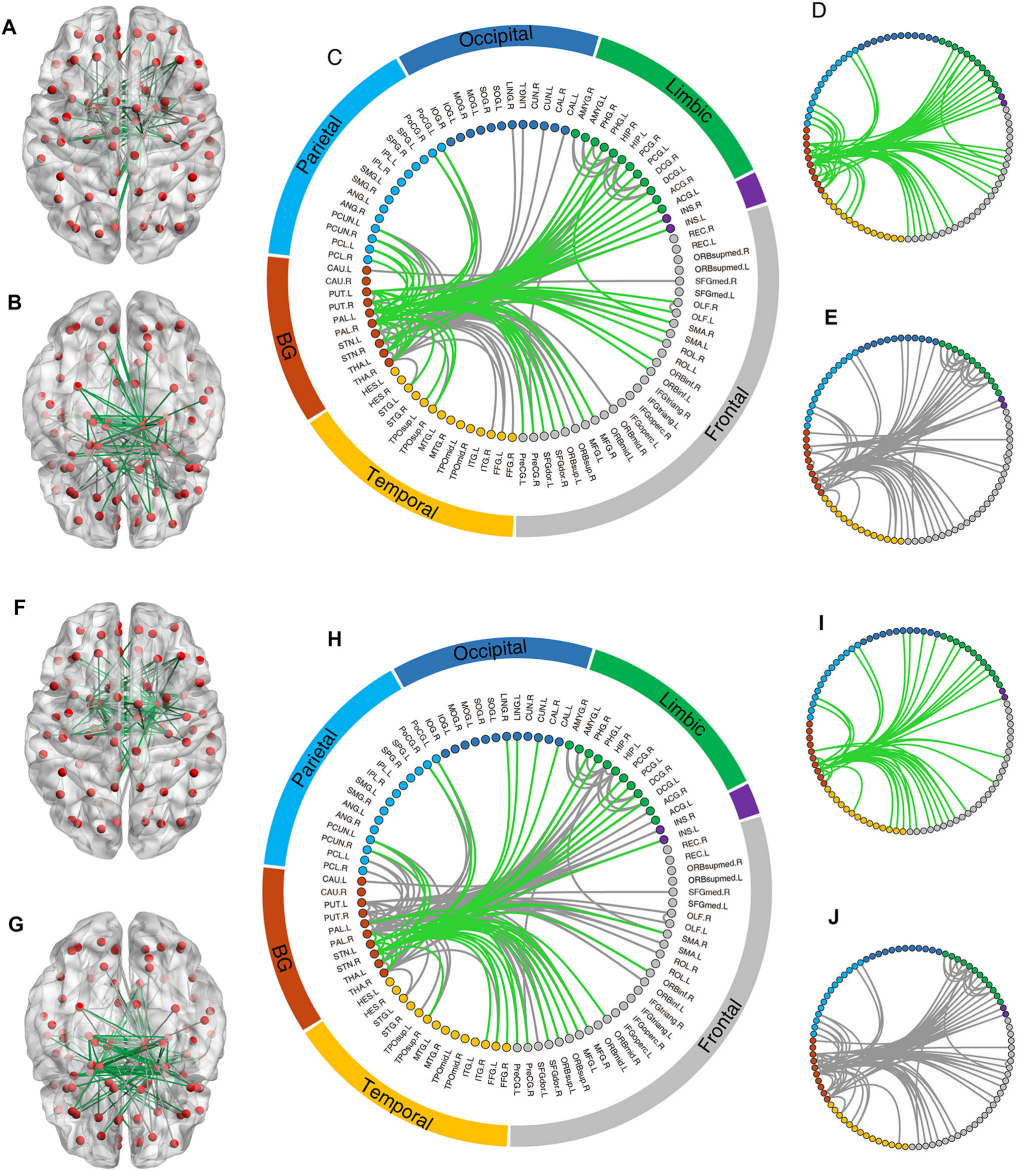
Representative topological changes in the functional brain connectome induced by stimulating the GP (**A**–**E**) and STN (**F**–**J**) in the local PD cohort. All involved brain regions (nodes, shown as red spheres) and connections (edges, shown as lines between nodes) were rendered on a transparent brain from superior (**A**) and inferior (**B**) axial views. As illustrated in **C**, all connections were categorized into different brain lobes. Green lines represent the removed abnormal connections that were significantly different between the local PD cohort and the HC group (*P* < 0.05 FWE corrected) before, but not after, neurostimulation. Grey lines represent the remaining abnormal connections after neurostimulation (*P* < 0.05 FWE corrected). **D**–**E** Altered connections indicated by green and grey lines in (**C**). **F**–**J** Topological changes of functional connections when stimulating the STN in the local PD cohort.

**Fig. 12:**
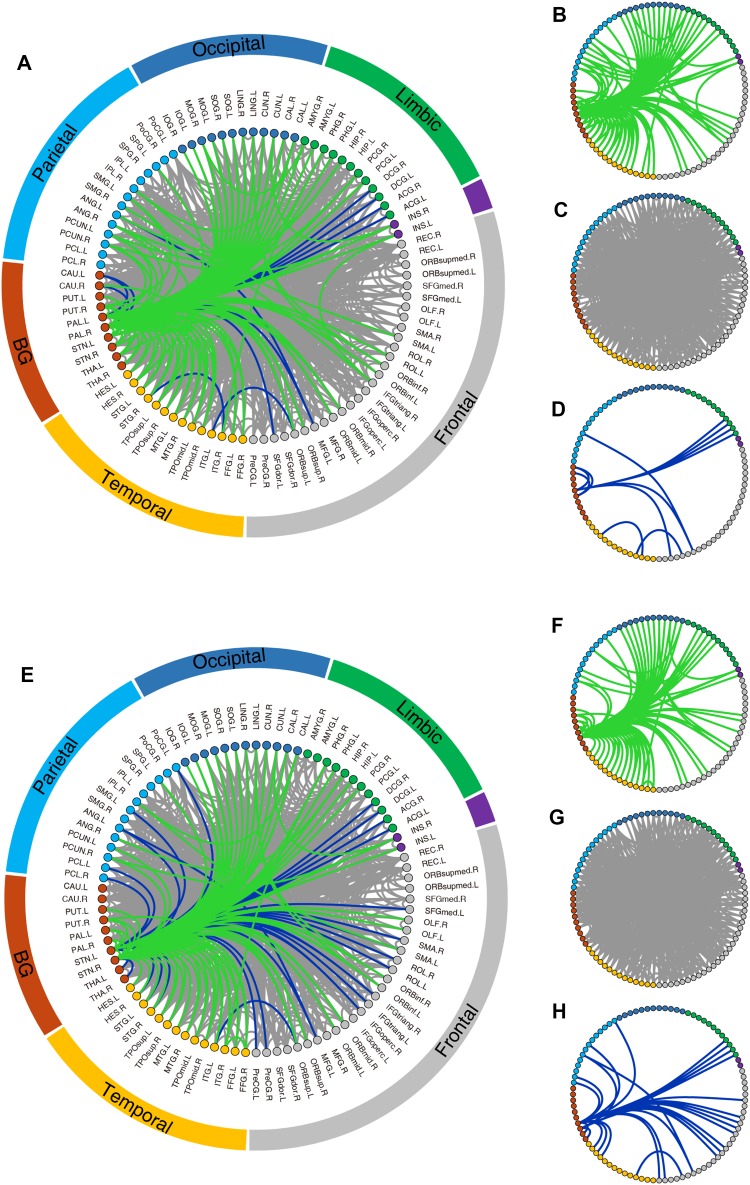
Topological changes in the functional brain connectome of the public PPMI PD group induced by neurostimulation targeting the GP (**A**–**D**) and STN (**E**–**H**). Green lines represent the removed abnormal connections that differ significantly between the PD patients and the HCs (*P* < 0.05, family-wise error (FWE) corrected) before, but not after neurostimulation. Blue lines represent the newly-emerged abnormal connections that became significantly different after neurostimulation (*P* < 0.05, FWE corrected). Gray lines represent the abnormal connections that persisted after neurostimulation (*P* < 0.05, FWE corrected). These three types of connections are illustrated together in the connection diagram (**A, E**) and separately in **B**–**D** and **F**–**H**.

We investigated whether and how focal stimulation of the GP or STN resulted in topological reconfiguration of the functional brain connectome in the local (Fig. 11) and public cohorts (Fig. 12). Neurostimulation remarkably resulted in the removal of a large number of abnormal connections between the target regions and widespread areas in the temporal, parietal, frontal, and limbic regions. Considerable abnormal connections in the basal ganglia circuits that bypass the GP (Fig. 12A, B) or STN (Fig. 12E, F) were eliminated in both the local and the public cohort, although a substantial number of connections emerged from the simulated intervention process for the public PD cohort. Taken together, these results demonstrate that focal stimulation leads to remarkable topological reconfiguration toward healthy bifurcation of the functional connectomes as measured in controls, suggesting potential therapeutic mechanisms for the alleviation of symptoms.

## Discussion

The present whole-brain modeling approach indeed describes a fundamental biological process with an analytic solution, and then translates the process to simulate the global effects of focal stimulation for clinically relevant purposes. It suggests that the spontaneous functional connectivity encoded in brain networks is sufficiently sensitive to capture an individual’s susceptibility in PD. Although thus far only substantiated using PD data, it can be readily generalized to other neuropsychiatric disorders and therapeutic stimulation modalities (e.g., transcranial magnetic stimulation), providing a general analytics whole-brain modeling framework for brain stimulation at the macroscale. Pioneering work based on the structural connectome or in combination with the functional connectome has shown promising results that model the dynamic network effects of a neural insult (lesion or stimulation), and predict network spreading and functional consequences [38, 49–53]. However, by assuming the repertoire of functional networks firmly rests upon the structural architecture of neuronal connections, this route is fundamentally bound to a basic understanding of the structure-function relationship [17, 23]. The present approach bypasses this difficulty and requires only the resting-state functional connectome to derive an empirical subject-specific transformation rule from the local to the global scale. This type of network diffusion process may stand for a fundamental process widely applicable to various biological systems, in that aberrant activity easily spreads through connected nodes and induces extensive pathological diffusions in the system. Moreover, we posit that reversal of abnormal network topology can indicate a positive outcome for brain stimulation therapy *in silico*, which formulates the fundamental basis for the predictions of pre-stimulation protocols in individuals, including candidate screening, target selection, and parameter tuning.

Target selection remains an unresolved challenge in the field of DBS in both neurological and psychiatric conditions [1, 3, 54, 55]. Insights gained from small animal experiments typically involve tedious procedures in which multiple brain regions are sequentially stimulated [56], and are not directly applicable as guidance for surgical procedures in patients [57]. Our resting-state connectivity-based approach was able to identify nuclei in the basal ganglia circuits as optimal targets in two independent cohorts, in striking accord with previous reports and clinical treatment routines for various movement disorders [5, 11, 12, 43, 48, 58, 59]. Nevertheless, we found a rather large proportion of patients (51.2% in the local cohort and 35.6% in the public cohort) for whom the best target was neither the GP nor the STN. In a very few patients (as shown in Fig. 8B), all the nuclei of the basal ganglia circuits ranked extremely low, suggesting that focal stimulation therapeutics may not be appropriate for them at all. Hence, our computational approach provides a more tailored strategy to guide individualized selection between these candidate targets, thereby improving the overall outcome of neurostimulation [5]. It has been shown that the thalamus is the best choice for stimulation in patients with severe tremor-related symptoms [60]. The putamen, another key component of the basal ganglia circuits, was identified among the top-ranking candidates, which is consistent with prior experience in patients [61]. Interestingly, the caudate emerged as a good candidate for some patients, especially in the public cohort (Fig. 10), despite its occurrence as a common target for stimulation in patients with epilepsy [55] or psychiatric conditions [3]. Treatment outcomes for the caudate had high variation that may lead to a relatively small effect. Surprisingly, the hippocampus was identified here as a potential target, most likely because it emerged as one of the key abnormal nodes when comparing the brain matrices of patients to those of controls. Accordingly, it is known to be pathologically involved in the non-motor symptoms of Parkinsonism [62]. However, it has not been reported hitherto in the clinical treatment of PD patients and future investigation in animals and human subjects is required to test the experimental outcomes of stimulation in this area.

Neurostimulation strength is another critical parameter affecting therapeutic outcomes in individuals. Determination of optimal strength in our modeling is analogous to the programming of a DBS device in individual patients, which often demands labor-intensive adjustment based on frequent symptom assessment by clinicians. In the present study, the priority of all regions as potential neurostimulation targets was ranked in each single patient when the optimal stimulation strength was applied to each area. Our identifications clearly showed that excessively strong or weak neurostimulation may not result in desirable modulation even at an appropriately selected target, and that optimal strengths (including up- or down-regulation) vary substantially among different targets across patients. These findings may serve as theoretical guidance for the tuning of stimulation protocols and possibly obviate the need for testing by trial-and-error on patients. This is a rather attractive premise, as brain stimulation with inappropriate parameters usually induces certain adverse effects and shortens battery life [2, 5]. More importantly, the present integrated strategy of identifying stimulation targets and strengths can be implemented on the basis of single patients, thereby opening a new avenue for the derivation of personalized treatments in phenomenologically inhomogeneous populations.

At the current stage, this connectome-based computational method has several practical limitations. First, the precise correspondence between the metrics (identified neurostimulation target and strength) of the computational model and the actual outcome of individual patients undergoing DBS with specific parameters (such as current amplitude and frequency of stimulus pulses) merits further *in vivo* experimental validation. Future retrospective or prospective clinical investigations in patients with DBS implantation would be invaluable for facilitating this translational application. Second, the precision of the present modeling relies on the parcellation of brain regions. As such, a delicately charted map [63, 64] would be preferred to provide executable guidance with greater precision, although it heightens the risk of increased model complexity and computation load. Thus, more insights into the biological validity of the present model using well-controlled animal studies [57] will help to balance this tradeoff.

Taken together, these findings, although exploratory in nature and requiring clinical validation, advance an unprecedented view of the global dynamics of brain function through the selective manipulation of a local area. This suggests that the ability to simulate and even control the perturbation of large-scale network dynamics evoked by focal manipulation may unfold a new dimension in the increasingly attractive field of brain connectomics [65, 66], with which the dynamic spread and functional consequences of pathological processes can be described. The development of neural circuit-based guidance approaches, coupling whole-brain connectomic modeling to clinical considerations of therapeutic intervention plans, which is yet to be established, may help to reach this goal and possibly diminish the risk of DBS-linked side-effects on cognitive function, mood, and behavior [15, 16, 67]. Stratification by a patient-specific functional connectome may have predictive value with respect to the efficacy of a neuromodulation treatment and even guide its choice, providing a stepping-stone for the advancement of translational progress in precision medicine.
